# Evaluation of Protein Cards: A nutrition education tool for metabolic bariatric surgery

**DOI:** 10.1371/journal.pone.0319235

**Published:** 2025-06-12

**Authors:** Patricia F. C. Acosta, Alexandra J. Heidl, Patricia M. Angeles, Biagina-Carla Farnesi, Peggy Alcindor, Angela S. Alberga, Julius Erdstein, Stephanie Saputra, Tamara R. Cohen

**Affiliations:** 1 Human Nutrition, Faculty of Land and Food Systems, The University of British Columbia, Vancouver, British Columbia, Canada; 2 Montreal Behavioural Medicine Centre, Centre Intégré Universitaire de Santé et de Services Sociaux du Nord de l’Ile de Montréal, Montréal, Quebec, Canada; 3 School of Human Nutrition, McGill University, Montréal, Quebec, Canada; 4 Centre of Excellence in Adolescent Severe Obesity, Montreal Children’s Hospital, McGill University Health Centre, Montréal, Quebec, Canada; 5 Department of Health, Kinesiology, and Applied Physiology, Concordia University, Montréal, Quebec, Canada; 6 Department of Mathematics and Statistics, Concordia University, Montréal, Quebec, Canada; Athens Medical Group, Psychiko Clinic, GREECE

## Abstract

**Background:**

Metabolic bariatric surgery (MBS) is a safe, effective treatment for severe obesity and its associated comorbidities. However, adherence to postoperative guidelines, particularly dietary protein intake remains a challenge. This study examined the suitability of the *Protein Cards*, a protein-focused nutrition education tool developed to support individuals meet their protein requirements during the postoperative diet stages: fluid, purée, soft, and regular.

**Methods:**

An online adapted version of the Suitability Assessment of Materials questionnaire was administered from September 2020 to May 2021. Participants were recruited via convenience sampling and advertisement. Participants rated the rated as “superior”, “adequate” or “not suitable” on content, literacy demand, graphic illustrations, layout and typography, learning stimulation and motivation, and cultural appropriateness, with scores of 2, 1, and 0, respectively.

**Results:**

A total of 437 individuals completed the online survey. Participants were identified as individuals who have undergone MBS (n = 263), caregivers of individuals who completed MBS (n = 68), and/or healthcare providers (including dietitians) specializing in MBS (n = 106). The *Protein Cards* received an overall “superior” rating of 73.00%. The tool had a high likelihood of use particularly for the soft diet stage (63.99 ± 20.70). Participants preferred the tool be available as a mobile application (63.41 ± 20.41) followed by paper book (59.40 ± 21.95) format.

**Conclusion:**

*Protein Cards* have the potential of supporting individuals who have undergone MBS in adopting healthy dietary habits, particularly in meeting their protein requirements. Future studies are needed to refine the *Protein Cards* and evaluate its usability among individuals post-MBS.

## Introduction

Severe obesity characterized by a body mass index (BMI) ≥35 kg/m^2^ affects ~1.9 million Canadian adults, [1] and is projected to double between 2020–2035 [2,3]. This significantly impacts health, overall quality of life, [4] and healthcare costs [5]. While lifestyle interventions remain the first line of treatment for obesity, [6,7] metabolic bariatric surgery (MBS) has been found to be a safe and effective treatment option for severe obesity and its associated comorbidities [8]. Common procedures, such as vertical sleeve gastrectomy and Roux-en-Y gastric bypass, modify the gastrointestinal tract to significantly reduce food intake and capacity to facilitate reduction of excess body weight and lower associated health risks [9]. Notable postoperative benefits include sustained weight reduction, improved cardiometabolic health and health-related quality of life, and remission of type 2 diabetes mellitus [10–14].

The long-term success of MBS remains dependent on commitment to lifestyle changes, particularly, food choices and eating behaviors that significantly influence MBS outcomes [15]. However, non-adherence to dietary guidelines postoperatively is frequently seen in the bariatric population [16–18]. Individuals report feeling overwhelmed [19] and uncertain about food choices [20]. Postoperative dietary guidelines include advancing through the various post-bariatric surgery diet stages: fluid, purée, soft, and regular diets [21,22]. The duration of each stage varies according to the patient’s tolerance, to ensure optimal healing of the gastrointestinal tract, minimize the risk of complications, and facilitate safe adaptation to foods [23].

One of the main nutritional concerns among patients post-MBS is inadequate dietary protein intake [24,25]. Inadequate protein intake has been demonstrated to result in greater loss of fat-free mass [26] and lean mass [27,28] while adequate protein intake postoperatively has been associated with increased satiety, weight loss, and improved body composition [27,29,30]. Current guidelines recommend 35% of total energy intake come from protein [31,32] or a minimum of 60 g/ day up to 1.5 g/kg ideal weight per day [21,24,33,34]. However, studies suggest that protein intake is often inadequate in this population, with a daily protein consumption of approximately 45 g [35,36]. In fact, protein deficiency remains the most severe macronutrient complication post-MBS, largely attributed to avoidance or aversion of protein-rich foods [24,37,38].

The use of nutrition education tools provide comprehensive and accessible information to increase knowledge and facilitate the adoption of healthy nutrition-related behaviors [39]. For these tools to be effective, they need be acceptable, suitable, and targeted to their end-users. However, there is a notable lack of tailored tools to help guide those who have undergone MBS in meeting their postoperative nutritional needs, particularly dietary protein intake. Majority of the materials have reading levels often exceeding what is appropriate for this demographic [40–44]. This is particularly concerning as health literacy was associated with successful weight loss among patients who have undergone MBS [45,46].

As such, the *“Protein Cards”* tool was developed to facilitate meeting the nutritional needs post-MBS, with emphasis on protein-rich dietary choices. The objective of this study was to evaluate the suitability of a patient nutrition education tool called *Protein Cards* among individuals who have undergone MBS, their caregivers, and healthcare providers working in MBS.

## Materials and methods

### Study design

This study was a cross-sectional, online survey conducted from September 2020 to May 2021 to assess perceptions and feedback on the suitability of the *Protein Cards*. The self-administered survey was designed to be completed within 20–30 minutes. Written informed consent was obtained from all individual participants included in the study. Irrespective of survey completion, participants were eligible to enter a draw for a chance to win a $50 CAD Amazon gift card. The study was approved by The University of British Columbia (Canada) Behavioural Research Ethics Board (ID: H20-01855).

### Recruitment and eligibility

#### Recruitment.

Registered dietitians working in MBS throughout Canada were identified using snowball and convenience sampling to increase feasibility of our recruitment efforts. Registered dietitians were contacted and asked to disseminate the study information (such as recruitment posters) to their patients. To further broaden reach and increase sample size, advertisements through Obesity Canada social media network were also used. The recruitment spanned from June 30, 2020 to April 30, 2021.

#### Eligibility.

Eligible participants included 1) individuals who have undergone MBS, 2) caregivers of individuals who have undergone MBS or, 3) healthcare providers (such as registered dietitians, nurses, physicians) working in MBS. Participants were excluded if they did not meet the eligibility criteria or if they could not read in English or French and lacked access to a device with Internet connection.

### Development of the nutrition education tool: *Protein Cards*

The development of *Protein Cards* was inspired by the dietitians at the Centre of Excellence in Adolescence Severe Obesity (CEASO, Montreal Children’s Hospital, Montreal, QC) and co-author Cohen. The tool is available in both English and French and includes 40 easy-to-follow recipes and food items, which were color coded to help guide patients meet their protein requirements for the various post-MBS diet stages: fluid(blue), purée (green), soft (pink), and regular (yellow). This tool focuses on recipes that are protein-rich, with a novel method of quantifying protein. Protein content was denoted in 10 g increments adapted from the carbohydrate counting methodology [47] in the form of a fish icon, which aims to simplify monitoring protein intake without complex calculations. The cards (n = 84) are double-sided: the front displayed the color-coded diet stage, an image of the completed recipe/food item, and protein content per serving while the back provided the recipe or food item along with tips for food substitutions, flavor enhancements, preparation, information on where to source ingredients, and food storage.

### Procedures

The self-administered survey was hosted through Qualtrics (Qualtrics International Inc., Seattle, Washington and Provo, Utah, United States), an online survey tool used for academic research at The University of British Columbia (Vancouver, British Columbia, Canada). The survey consisted of two parts: the first section included sociodemographic questions, and the second section utilized an adapted version of the Suitability Assessment of Materials (SAM) questionnaire to evaluate the content and suitability of chosen pages of the *Protein Cards*, which included the cover page, instruction page, and four representative recipes/ food items corresponding to each diet stage post-surgery.

### Measures

#### Suitability Assessment of Materials (SAM).

The suitability of *Protein Cards* was evaluated using SAM, a widely used and validated rating tool to assess the appropriateness of health information materials [48–51]. All questions from the 21-item SAM questionnaire were adapted to modify the wording to accommodate varying literacy levels. These questions were classified into six categories: content, literacy demand, graphic illustrations, layout and typography, learning stimulation and motivation, and cultural appropriateness. An additional two questions were included in the survey to understand the usability of the cards [“How likely would you consult the recipe book during the following periods: fluid, purée, soft and regular diet?”] and to determine the preferred delivery format [“How likely would you use the recipe book in the following formats: paper book, electronic book, mobile application, and webpage?”].

Participants were asked to assess the *Protein Cards* using three possible ratings for each question: “superior”, “adequate”, or “not suitable”, which were assigned point scores of 2, 1, and 0, respectively. The total SAM score was calculated as the sum of earned points divided by the total maximum score of 42. The final SAM scores were reported as percentages, with 70–100% indicating superior material, 40–69% adequate material, and 0–39% not suitable material. Participants rated the usability of the cards across MBS diet stages and the preferred delivery format on a scale of 0–100 (0 = not at all; 100 = highly).

### Data analysis

Survey results were obtained in TSV format and all analyses were done with R version 4.3.1 (The R Foundation for Statistical Computing, Vienna, Austria), with p < 0.05 considered statistically significant. Descriptive statistics were used to report participant characteristics and SAM assessment scores by category, as total counts and percentages. Summary statistics of the likelihood of *Protein Cards* use across MBS diet stages and its preferred method of delivery were reported as means and standard deviations. Analyses were repeated independently for patients, healthcare providers, and caregivers, to gain a better understanding of the participants’ preferences. All data were examined for normality. Chi-square tests or Fisher’s exact tests were used to assess categorical differences in SAM scores across participant groups. The Kruskal –Wallis test was used to compare responses across participant groups for likelihood of use across MBS diet stages and preferred delivery formats.

## Results

### Participant characteristics

Of the 1,836 surveys received, 437 survey responses were included in the analysis. Participants were excluded if they did not meet inclusion criteria (n = 237), absence of consent (n = 1), missing/ incomplete surveys (n = 177) and data validity, including recurring email and IP addresses (n = 842), inappropriate email address (n = 1), duplicated written responses (n = 33), and completion time under five minutes (n = 103) and participants <19 years (n = 5). Among the 437 participants, 263 (60.18%) were individuals who have undergone MBS, 106 (24.26%) were healthcare providers working in MBS, and 68 (15.56%) were caregivers of patients who had MBS. Majority of participants were female (62.70%) aged between 29–39 (80.92%), self-identified as White (59.17%), completed college/ university educational level (31.65%) and reported an individual annual income of $45,000- $74,999 CAD (44.16%) (Table 1).

**Table 1:** Characteristics of survey participants, n=437.

Characteristics	Count (%)
Age	
<29	154 (35.40)
30-39	198 (45.52)
40-49	57 (13.10)
50-59	24 (5.52)
60-69	2 (0.46)
Not specified	2 (---)
Sex	
Male	163 (37.30)
Female	274 (62.70)
Ethnicity	
Aboriginal/ First Nations/ Métis	63 (14.45)
Arab	2 (0.46)
Black	48 (11.00)
Asian	
Chinese	11 (2.52)
Filipino	2 (0.46)
South Asian (East Indian, Pakistani, Sri Lankan, etc.)	24 (5.50)
West Asian (Iranian, Afghan, etc.)	1 (0.23)
Latin American	9 (2.06)
White	258 (59.17)
Mixed	18 (3.92)
Not specified	1 (---)
Occupation	
Patients	263 (60.18)
Healthcare providers	
Dietitians	78 (17.85)
Others	28 (6.41)
Caregivers	68 (15.56)
Education level	
Elementary school	23 (5.28)
High school	49 (11.24)
CEGEP	208 (47.71)
Vocational school or apprenticeship training	17 (3.90)
College	75 (17.20)
University	63 (14.45)
Prefer to not answer	1 (0.23)
Not applicable	1 (---)
Income	
Less than $15,000	27 (6.18)
$15,000 - $29,000	57 (13.04)
$29,000 - $44,999	74 (16.93)
$45,000 - $59,999	100 (22.88)
$60,000 - $74,999	93 (21.28)
$75,000 or more	81 (18.54)
Prefer to not answer	5 (1.14)
Not specified	---

### SAM questionnaire

The *Protein Cards* tool was scored as a “superior” postoperative nutrition education tool for MBS, with an overall rating of 73.00%. The six SAM categories received superior mean score values: content (73.90%), literacy demand (72.00%), graphic illustrations (74.50%), layout and typography (71.80%), learning stimulation and motivation (73.50%), and cultural appropriateness (72.10%), which significantly differed by category (p < 0.0001) (Table 2). When examining the data by participant groups, patients significantly gave higher ratings for literacy demand (74.56%, p = 0.009), graphic illustrations (76.62%, p < 0.001), learning stimulation and motivation (75.24%, p = 0.02). Content and cultural appropriateness scores did not significantly differ across participants groups (p = 0.07; p = 0.7, respectively) (Table 3).

**Table 2:** Suitability Assessment of Materials (SAM) scores by category.

SAM scores by category	Count (%) of participants scoring material as:			Missing	Mean score (%)^1^
	Superior (2)	Adequate (1)	Not suitable (0)		
**Content**					**4.43 (73.9%)**
Purpose	264 (61.40%)	164 (38.14%)	2 (0.47%)	7	1.61 (80.5%)
Content Topics	203 (47.21%)	216 (50.23%)	11 (2.56%)	7	1.45 (72.3%)
Scope	215 (50.00%)	194 (45.12%)	21 (4.88%)	7	1.45 (72.6%)
**Literacy demand**					**7.19 (72.0%)**
Reading grade level	152 (34.86%)	257 (58.94%)	27 (6.19%)	1	1.29 (64.3%)
Writing style	247 (56.52%)	185 (42.3%)	5 (1.14%)	0	1.55 (77.7%)
Vocabulary	223 (51.15%)	199 (45.64%)	14 (3.21%)	1	1.48 (74.0%)
Sentence construction	205 (46.91%)	217 (49.66%)	15 (3.43%)	0	1.43 (71.7%)
Organization	214 (48.97%)	204 (46.68%)	19 (4.35%)	0	1.45 (72.3%)
**Graphic illustrations**					**5.96 (74.5%)**
Book cover	262 (60.23%)	158 (36.32%)	15 (3.45%)	2	1.57 (78.4%)
Type of illustrations	204 (46.79%)	215 (49.31%)	17 (3.90%)	1	1.43 (71.4%)
Relevance of illustrations	256 (58.72%)	155 (35.55%)	25 (5.73%)	1	1.53 (76.5%)
Captions	205 (46.91%)	223 (51.03%)	9 (2.06%)	0	1.45 (72.4%)
**Layout and typography**					**4.31 (71.8%)**
Typography	219 (50.23%)	206 (47.25%)	11 (2.52%)	1	1.48 (73.9%)
Layout	187 (42.89%)	237 (54.36%)	12 (2.75%)	1	1.40 (70.1%)
Subheadings and “chunking”	206 (47.14%)	216 (49.43%)	15 (3.43%)	0	1.44 (71.9%)
**Learning stimulation and motivation**					**5.88 (73.5%)**
Interaction	208 (47.93%)	215 (49.54%)	11 (2.53%)	3	1.45 (72.7%)
Desired behavior patterns	220 (50.34%)	213 (48.74%)	4 (0.92%)	0	1.49 (74.9%)
Motivation (use of Fish icon)	242 (55.38%)	174 (39.82%)	21 (4.81%)	0	1.51 (75.3%)
Confidence in task completion	203 (46.56%)	221 (50.69%)	12 (2.75%)	1	1.44 (71.9%)
**Cultural appropriateness**					**2.88 (72.1%)**
Cultural match	214 (48.97%)	214 (48.97%)	9 (2.06%)	0	1.47 (73.5%)
Cultural image and examples	191 (43.71%)	236 (54.00%)	10 (2.29%)	0	1.41 (70.7%)
**Total score** ^2^					**30.65 (73.00%)**

^1^Percentages shown are the item’s mean divided by the maximum value of the score. (e.g., $\;\%\;Content=\;\frac{Mean\;(Purpose+Content\;topics+Scope)}{6\;}$).

^2^Total score’s percentage was calculated by dividing the average of total score by 42.

**Table 3:** Overall SAM scores among participants.

SAM scores by category (overall)	Count (%) of participants’ overall evaluation^1^			Mean score (%)^2^	p-value^3^
	Not suitable	Adequate	Superior		
Content					
Patients	9 (3.42)	102 (38.78)	152 (57.79)	4.52 (75.41%)	0.07
Dietitians	5 (6.41)	42 (53.85)	31 (19.74)	4.08 (67.95%)	
Healthcare providers	1 (3.57)	15 (53.57)	12 (42.86)	4.21 (70.24%)	
Caregivers	1 (1.47)	29 (42.65)	38 (55.88)	4.59 (76.47%)	
Literacy demand					
Patients	2 (0.76)	70 (26.62)	191 (72.62)	7.46 (74.56%)	0.009
Dietitians	–	36 (46.15)	42 (53.85)	6.68 (66.80%)	
Healthcare providers	1 (3.57)	8 (28.57)	19 (67.86)	6.64 (66.43%)	
Caregivers	–	27 (39.71)	41 (60.29)	7.00 (70.00%)	
Graphic and illustrations					
Patients	5 (1.90)	64 (24.33)	194 (73.76)	6.13 (76.62%)	0.001
Dietitians	7 (8.97)	28 (35.90)	43 (55.13)	5.56 (69.55%)	
Healthcare providers	1 (3.57)	14 (50.00)	13 (46.43)	5.54 (69.20%)	
Caregivers	3 (4.41)	21 (30.88)	44 (64.71)	5.94 (74.27%)	
Layout and typography					
Patients	3 (1.14)	148 (56.27)	112 (42.59)	4.35 (72.43%)	0.002
Dietitians	2 (2.56)	51 (65.38)	25 (32.05)	4.08 (67.95%)	
Healthcare providers	–	20 (71.43)	8 (28.57)	3.96 (66.07%)	
Caregivers	3 (4.41)	24 (35.29)	41 (60.29)	4.57 (76.23%)	
Learning stimulation and motivation					
Patients	2 (0.76)	79 (30.04)	182 (69.20)	6.02 (75.24%)	0.02
Dietitians	4 (5.13)	35 (44.87)	39 (50.00)	5.51 (68.91%)	
Healthcare providers	1 (3.57)	10 (35.71)	17 (60.71)	5.64 (70.54%)	
Caregivers	–	22 (32.35)	46 (67.65)	5.85 (73.16%)	
Cultural appropriateness					
Patients	4 (1.52)	79 (30.04)	180 (68.44)	2.91 (72.81%)	0.7
Dietitians	3 (3.85)	24 (30.77)	51 (65.38)	2.80 (69.87%)	
Healthcare providers	1 (3.57)	8 (28.57)	19 (67.86)	2.93 (73.21%)	
Caregivers	3 (4.41)	20 (29.41)	45 (66.18)	2.85 (71.32%)	

^1^In the overall evaluation, 0–39% is considered ‘not suitable’, 40–69% is ‘adequate’ and 70% is ‘superior’. We showed the number of participants that falls under each category by dividing their overall score with the maximum score of the category.

^2^Mean score is the average of total score and the percentage was calculated by dividing the mean with the maximum score of the category.

^3^p-value was calculated through Fisher’s exact test to compare SAM scores between patients, dietitians, healthcare providers, and caregivers. p < 0.05 is considered statistically significant.

Abbreviation used: SAM; Suitability Assessment of Materials

Participants reported the highest likelihood of using *Protein Cards* during the soft diet stage (63.99 ± 20.70) followed by regular diet (63.78 ± 21.05), purée diet (58.58 ± 22.16), and full fluid diet (57.62 ± 21.13), which significantly differed across diet stages (p < 0.001) and participant groups (p < 0.001) (Table 4). When considering the preferred delivery format of *Protein Cards*, mobile application (63.41 ± 20.41) was found to be the most favored option, with highest ratings among caregivers (68.47 ± 19.51), healthcare providers (64.00 ± 23.93), patients (64.08 ± 19.17), and dietitians (56.44 ± 22.45) (p = 0.007). Preferences for the webpage format did not significantly differ across participants groups. (Table 5).

**Table 4:** Likelihood of Protein Cards use across diet progression stages of metabolic bariatric surgery among patients, healthcare providers and caregivers.

Diet Stage	*n*	Mean ± SD	p-value^1^
**Full fluid diet**	435	57.62 ± 21.13	
Patients		58.66 ± 21.45	<0.001
Healthcare providers Dietitian Others		53.68 ± 20.76 58.57 ± 21.61	
Caregivers		57.66 ± 20.02	
**Purée diet**	436	58.58 ± 22.16	
Patients		61.89 ± 20.65	<0.001
Healthcare providers Dietitian Others		49.96 ± 22.68 59.11 ± 24.75	
Caregivers		55.32 ± 23.46	
**Soft diet**	434	63.99 ± 20.70	
Patients		66.31 ± 19.32	<0.001
Healthcare providers Dietitian Others		57.37 ± 21.32 61.64 ± 25.19	
Caregivers		63.41 ± 21.88	
**Regular diet**	435	63.78 ± 21.05	
Patients		65.52 ± 19.22	<0.001
Healthcare providers Dietitian Others		54.51 ± 23.95 64.04 ± 25.07	
Caregivers		67.50 ± 20.06	

^1^p-value was calculated through Kruskal-Wallis test to compare likelihood of use across patients, healthcare providers, and caregivers for each diet stage.

**Table 5:** Preferred delivery format of *Protein Cards* among patients, healthcare providers and caregivers.

Format	*n*	Mean ± SD	p-value^1^
**Paper book**	435	59.40 ± 21.95	
Patients		62.16 ± 20.62	0.008
Healthcare providers Dietitian Others		54.16 ± 22.74 55.57 ± 22.43	
Caregivers		56.24 ± 24.45	
**Electronic book**	435	56.32 ± 21.00	
Patients		57.90 ± 21.42	0.002
Healthcare providers Dietitian Others		48.62 ± 19.46 53.82 ± 17.28	
Caregivers		59.97 ± 20.64	
**Mobile application**	434	63.41 ± 20.41	
Patients		64.08 ± 19.17	0.007
Healthcare providers Dietitian Others		56.44 ± 22.45 64.00 ± 23.93	
Caregivers		68.47 ± 19.51	
**Webpage**	436	59.34 ± 22.55	
Patients		61.94 ± 21.65	0.07
Healthcare providers Dietitian Others		54.79 ± 21.94 53.29 ± 25.66	
Caregivers		56.94 ± 24.22	

^1^p-value was calculated through Kruskal-Wallis test to compare responses from patients, healthcare providers, and caregivers according to the delivery format preferences.

## Discussion

The current study assessed the suitability of *Protein Cards*, a postoperative patient nutrition education tool in a sample of individuals who have undergone MBS, their caregivers, and healthcare providers working in MBS. Research suggests that there is a lack of diversity of nutrition education materials tailored for the needs of individuals who undergo MBS [42,52]. The focus of *Protein Cards* is to provide easy-to-follow recipes that are protein-rich to facilitate healthy dietary habits and meet protein requirements during the post-MBS diet stages.

Creating suitable dietary education tools for the intended audience is critical to support the adoption of healthy dietary behavior change. Participants reported *Protein Cards* to be a “superior” tool with an overall rating of 73.00%, which aligns or exceeds the ratings of other studies that assessed health educational materials [49–51]. The *Protein Cards* received a superior rating for content- purpose (80.5%), indicating participants’ clear understanding of the tool’s objectives in addressing the dietary needs, particularly protein intake following MBS. Additionally, such a rating suggests the tool’s suitability in supporting optimal transition through the various diet stages and in facilitating the adoption of healthy dietary habits.

Different aspects of nutrition education resources need to be considered to ensure the tool’s suitability. One of those is the tool’s readability. In this study, participants rated the readability of *Protein Cards* to be between sixth to eighth grade reading level, which was likely due to the use of plain language. Other studies have assessed the readability of MBS patient materials and found them to be overall of poor quality; these materials [40,41,43,44] were above the recommended sixth grade reading level. In a specific examination of patient information publications from the American Society of Metabolic and Bariatric Surgery, Massie et al. (2024) utilized multiple validated assessment tools to evaluate the readability of the publications. Out of the n = 11 publications analyzed, none met the recommended sixth grade reading level, suggesting refinements are needed. Given these findings, there is significant opportunity to enhance the reading grade level of *Protein Cards*, ensuring the use of clear and plain language to better support patients in meeting their protein needs post-MBS.

Researchers who work in the area of developing nutrition education tools should consider the preferred delivery format to enhance accessibility and user satisfaction of these educational tools. The likelihood of participants utilizing the *Protein Cards* varied based on delivery format, including paper book, electronic book, mobile application, and webpage. Participants noted a high preference for a mobile application, which has been postulated to reach and gain more engagements among individuals [53]. This is consistent with findings of studies that explored adults’ perspectives on patient health education materials, which have shown a preference for varied types of electronic and paper resources [54,55].

This highlights that the *Protein Cards* might be more accessible for a diverse sample of patients if they are available in diverse delivery formats.

MBS is a complex process the extends beyond the surgery itself, involving not only the patients, but also their healthcare providers and caregivers to ensure long-term success of dietary management. Our study addresses this by concurrently evaluating the suitability of a nutrition education tool among these groups, with the aim to refine the tool to make it more comprehensive, suitable, and practical for implementation in clinical practice. Research demonstrate that patients can meet nutritional needs with healthy food choices, [56] but experience challenges adhering to postoperative guidance. This suggests that lifelong support is needed, which may lead to improved self-efficacy, a hallmark for behavioral compliance [57]. Additionally, social support from family and friends has been shown to significantly influence dietary management, [57,58] physical and psychological well-being, [58,59] by facilitating effective coping strategies for stressors – a need that becomes more critical as patients adjust to their “new normal” post-MBS [60]. In a study that explored experiences of partners of individuals who have undergone MBS found that they faced various changes, including their level of engagement with the MBS process, such as efforts to access resources available to have a better understanding of the surgical process, and adoption to behavioral changes made by their spouses [61]. While another study highlighted that healthcare providers working in Canadian bariatric surgery program experienced challenges in making clinical decisions on how to best guide patients regarding nutrition pre- and post-surgery [62]. These demonstrated the ongoing need for bariatric resources that are user-friendly, easily accessible, and adaptable to different patient profiles. These findings convey the importance of a patient-centered approach that considers patients, their caregivers, and healthcare providers as integral stakeholders in MBS.

The strengths of this study include a large sample size, and the diverse engagement of stakeholders (patients, caregivers, and healthcare providers) provided a multifaceted evaluation of the *Protein Cards*. We also used SAM, an evaluation questionnaire that surveys on different aspects of patient education materials. However, this study has limitations, including the lack of data on the type of MBS, which could provide further insights into the tailored nutritional needs of patients. Moreover, recruitment methods used may limit the generalizability of the findings.

In summary, there is a critical need for clear, accessible nutrition education tools tailored for MBS. Our findings demonstrate that the tool we have developed, *Protein Cards*, is usable and suggests potential benefits for meeting protein requirements across the various stages of post-MBS diet. Participants found the tool to be superior, with a high likelihood of use during these stages. Such tools are essential for supporting patients navigate this complex journey and are pivotal in advancing bariatric care. This can aid in facilitating a smoother transition to a broader range of foods and assist with the emotional and psychological challenges associated with dietary intake post-MBS. Future work will involve refining the tool and pilot testing the tool in clinical settings with individuals undergoing MBS, which will inform a future larger intervention trial aimed at evaluating the efficacy of *Protein Cards* in helping meet protein recommendations post-MBS.

## References

[1] Twells LK, Janssen I, Kuk JL. Canadian Adult Obesity Clinical Practice Guidelines: Epidemiology of Adult Obesity. Available from:https://obesitycanada.ca/guidelines/epidemiology.

[2] Koliaki C, Dalamaga M, Liatis S. Update on the obesity epidemic: after the sudden rise, is the upward trajectory beginning to flatten? Curr Obes Rep. 2023;12(4):514–27. doi: 10.1007/s13679-023-00527-y 37779155 PMC10748771

[3] Finkelstein EA, Khavjou OA, Thompson H, Trogdon JG, Pan L, Sherry B, et al. Obesity and severe obesity forecasts through 2030. Am J Prev Med. 2012;42(6):563–70. doi: 10.1016/j.amepre.2011.10.026 22608371

[4] Chang C-Y, Hung C-K, Chang Y-Y, Tai C-M, Lin J-T, Wang J-D. Health-related quality of life in adult patients with morbid obesity coming for bariatric surgery. Obes Surg. 2010;20(8):1121–7. doi: 10.1007/s11695-008-9513-z 18463932 PMC2910893

[5] Butalia S, Luu H, Guigue A, Martins KJB, Williamson T, Klarenbach SW. Health care cost of severe obesity and obesity-related comorbidities: a retrospective cohort study from Alberta, Canada. Obes Res Clin Pract. 2023;17(5):421–7. 37709630 10.1016/j.orcp.2023.09.006

[6] Hassan Y, Head V, Jacob D, Bachmann MO, Diu S, Ford J. Lifestyle interventions for weight loss in adults with severe obesity: a systematic review. Clin Obes. 2016;6(6):395–403. doi: 10.1111/cob.12161 27788558

[7] Haywood C, Sumithran P. Treatment of obesity in older persons-A systematic review. Obes Rev. 2019;20(4):588–98. doi: 10.1111/obr.12815 30645010

[8] Eisenberg D, Shikora SA, Aarts E, Aminian A, Angrisani L, Cohen RV, et al. 2022 American Society of Metabolic and Bariatric Surgery (ASMBS) and International Federation for the Surgery of Obesity and Metabolic Disorders (IFSO) Indications for Metabolic and Bariatric Surgery. Obes Surg. 2023;33(1):3–14. doi: 10.1007/s11695-022-06332-1 36336720 PMC9834364

[9] Nguyen NT, Varela JE. Bariatric surgery for obesity and metabolic disorders: state of the art. Nat Rev Gastroenterol Hepatol. 2017;14(3):160–9. doi: 10.1038/nrgastro.2016.170 27899816

[10] Pipek LZ, Moraes WAF, Nobetani RM, Cortez VS, Condi AS, Taba JV, et al. Surgery is associated with better long-term outcomes than pharmacological treatment for obesity: a systematic review and meta-analysis. Sci Rep. 2024;14(1):9521. 38664450 10.1038/s41598-024-57724-5 PMC11045962

[11] Arterburn DE, Telem DA, Kushner RF, Courcoulas AP. Benefits and risks of bariatric surgery in adults: a review. JAMA. 2020;324(9):879–87. 32870301 10.1001/jama.2020.12567

[12] Konttinen H, Sjöholm K, Carlsson LMS, Peltonen M, Svensson PA. Fifteen-year changes in health-related quality of life after bariatric surgery and non-surgical obesity treatment. Int J Obes. 2024;48(10):1447–56. 10.1038/s41366-024-01572-w PMC11420074 38902388

[13] Courcoulas AP, Daigle CR, Arterburn DE. Long term outcomes of metabolic/bariatric surgery in adults. BMJ. 2023;383:e071027. 10.1136/bmj-2022-071027 38110235

[14] Affinati AH, Esfandiari NH, Oral EA, Kraftson AT. Bariatric surgery in the treatment of type 2 diabetes. Curr Diab Rep. 2019;19(12):156. 31802258 10.1007/s11892-019-1269-4 PMC7522929

[15] Cheung HC, Strodl E, Musial J, MacLaughlin HL, Byrnes A, Lewis C-A, et al. Associations between diet composition, dietary pattern, and weight outcomes after bariatric surgery: a systematic review. Int J Obes (Lond). 2023;47(9):764–90. doi: 10.1038/s41366-023-01333-1 37407830 PMC10439005

[16] Hood MM, Corsica J, Bradley L, Wilson R, Chirinos DA, Vivo A. Managing severe obesity: understanding and improving treatment adherence in bariatric surgery. J Behav Med. 2016;39(6):1092–103. 27444752 10.1007/s10865-016-9772-4

[17] Hood MM, Kelly MC, Feig EH, Webb V, Bradley LE, Corsica J. Measurement of adherence in bariatric surgery: a systematic review. Surg Obes Relat Dis. 2018;14(8):1192–201. doi: 10.1016/j.soard.2018.04.013 29853195

[18] Sundgot-Borgen C, Bond DS, Rø Ø, Sniehotta F, Kristinsson J, Kvalem IL. Associations of adherence to physical activity and dietary recommendations with weight recurrence 1–5 years after metabolic and bariatric surgery. Surg Obes Relat Dis. 2024;20(4):383–90. 38160134 10.1016/j.soard.2023.11.014

[19] Suvarnakar A, Hose BZ, Busog DN, McCloud S, Chao GF, Miller K. Falling short in bariatric surgery: an exploration of key barriers and motivators of attrition. Am J Surg. 2024;115827. 39029267 10.1016/j.amjsurg.2024.115827

[20] Tolvanen L, Christenson A, Bonn SE, Surkan PJ, Lagerros YT. Patients’ perspectives on dietary patterns and eating behaviors during weight regain after gastric bypass surgery. Obes Surg. 2023;33(8):2517–26. doi: 10.1007/s11695-023-06718-9 37402891 PMC10345057

[21] Mechanick JI, Apovian C, Brethauer S, Garvey WT, Joffe AM, Kim J, et al. Clinical practice guidelines for the perioperative nutrition, metabolic, and nonsurgical support of patients undergoing bariatric procedures - 2019 update: cosponsored by American Association of Clinical Endocrinologists/American College of Endocrinology, The Obesity Society, American Society for Metabolic & Bariatric Surgery, Obesity Medicine Association, and American Society of Anesthesiologists. Surg Obes Relat Dis. 2020;16(2):175–247. doi: 10.1016/j.soard.2019.10.025 31917200

[22] Handzlik-Orlik G, Holecki M, Orlik B, Wyleżoł M, Duława J. Nutrition management of the post-bariatric surgery patient. Nutr Clin Pract. 2015;30(3):383–92. doi: 10.1177/0884533614564995 25547336

[23] Bettini S, Belligoli A, Fabris R, Busetto L. Diet approach before and after bariatric surgery. Rev Endocr Metab Disord. 2020;21(3):297–306. doi: 10.1007/s11154-020-09571-8 32734395 PMC7455579

[24] Sherf Dagan S, Goldenshluger A, Globus I, Schweiger C, Kessler Y, Kowen Sandbank G. Nutritional recommendations for adult bariatric surgery patients: clinical practice. Adv Nutr. 2017;8(2):382–94. 28298280 10.3945/an.116.014258 PMC5347111

[25] Steenackers N, Gesquiere I, Matthys C. The relevance of dietary protein after bariatric surgery: what do we know? Curr Opin Clin Nutr Metab Care. 2018;21(1):58–63. doi: 10.1097/MCO.0000000000000437 29035973

[26] Sherf Dagan S, Tovim TB, Keidar A, Raziel A, Shibolet O, Zelber-Sagi S. Inadequate protein intake after laparoscopic sleeve gastrectomy surgery is associated with a greater fat free mass loss. Surg Obes Relat Dis. 2017;13(1):101–9. doi: 10.1016/j.soard.2016.05.026 27521254

[27] Ito MK, Gonçalves VSS, Faria SLCM, Moizé V, Porporatti AL, Guerra ENS, et al. Effect of protein intake on the protein status and lean mass of post-bariatric surgery patients: a systematic review. Obes Surg. 2017;27(2):502–12. doi: 10.1007/s11695-016-2453-0 27844254

[28] Raftopoulos I, Bernstein B, O’Hara K, Ruby JA, Chhatrala R, Carty J. Protein intake compliance of morbidly obese patients undergoing bariatric surgery and its effect on weight loss and biochemical parameters. Surg Obes Relat Dis. 2011;7(6):733–42. 21925961 10.1016/j.soard.2011.07.008

[29] van den Broek M, de Heide LJM, Veeger NJGM, van der Wal-Oost AM, van Beek AP. Influence of dietary protein and its amino acid composition on postoperative outcomes after gastric bypass surgery: a systematic review. Nutr Rev. 2016;74(12):749–73. doi: 10.1093/nutrit/nuw042 27864536

[30] Faria SL, Faria OP, Buffington C, de Almeida Cardeal M, Ito MK. Dietary protein intake and bariatric surgery patients: a review. Obes Surg. 2011;21(11):1798–805. doi: 10.1007/s11695-011-0441-y 21590346

[31] Mechanick JI, Youdim A, Jones DB, Garvey WT, Hurley DL, McMahon MM. Clinical practice guidelines for the perioperative nutritional, metabolic, and nonsurgical support of the bariatric surgery patient--2013 update: cosponsored by American Association of Clinical Endocrinologists, The Obesity Society, and American Society for Metabolic & Bariatric Surgery. Obes Silver Spring Md. 2013;21(Suppl 1):S1–27. 10.1002/oby.20461 PMC4142593 23529939

[32] Moizé VL, Pi-Sunyer X, Mochari H, Vidal J. Nutritional pyramid for post-gastric bypass patients. Obes Surg. 2010;20(8):1133–41. doi: 10.1007/s11695-010-0160-9 20401543

[33] Stenberg E, Dos Reis Falcão LF, O’Kane M, Liem R, Pournaras DJ, Salminen P, et al. Guidelines for perioperative care in bariatric surgery: Enhanced Recovery After Surgery (ERAS) society recommendations: a 2021 update. World J Surg. 2022;46(4):729–51. doi: 10.1007/s00268-021-06394-9 34984504 PMC8885505

[34] O’Kane M, Parretti HM, Pinkney J, Welbourn R, Hughes CA, Mok J, et al. British Obesity and Metabolic Surgery Society Guidelines on perioperative and postoperative biochemical monitoring and micronutrient replacement for patients undergoing bariatric surgery-2020 update. Obes Rev. 2020;21(11):e13087. doi: 10.1111/obr.13087 32743907 PMC7583474

[35] Moize V, Geliebter A, Gluck ME, Yahav E, Lorence M, Colarusso T. Obese patients have inadequate protein intake related to protein intolerance up to 1 year following Roux-en-Y gastric bypass. Obes Surg. 2003;13(1). 10.1381/096089203321136548 12630609

[36] Verger EO, Aron-Wisnewsky J, Dao MC, Kayser BD, Oppert J-M, Bouillot J-L, et al. Micronutrient and protein deficiencies after gastric bypass and sleeve gastrectomy: a 1-year follow-up. Obes Surg. 2016;26(4):785–96. doi: 10.1007/s11695-015-1803-7 26205215

[37] Lupoli R, Lembo E, Saldalamacchia G, Avola CK, Angrisani L, Capaldo B. Bariatric surgery and long-term nutritional issues. World J Diabetes. 2017;8(11):464–74. 29204255 10.4239/wjd.v8.i11.464 PMC5700383

[38] Ben-Porat T, Lahav Y, Cohen TR, Bacon SL, Buch A, Moizé V. Is there a need to reassess protein intake recommendations following metabolic bariatric surgery?. Current Obesity Reports. 2025;14(1):15. 39878797 10.1007/s13679-025-00607-1 PMC11779789

[39] Contento IR. Nutrition education: linking research, theory, and practice. Asia Pac J Clin Nutr. 2008;17 Suppl 1:176–9. 18296331

[40] Gutterman SA, Schroeder JN, Jacobson CE, Obeid NR, Suwanabol PA. Examining the accessibility of online patient materials for bariatric surgery. Obes Surg. 2023;33(3):975–7. doi: 10.1007/s11695-022-06440-y 36602722

[41] Lucy AT, Rakestraw SL, Stringer C, Chu D, Grams J, Stahl R, et al. Readability of patient education materials for bariatric surgery. Surg Endosc. 2023;37(8):6519–25. doi: 10.1007/s00464-023-10153-3 37277519

[42] Wang YN, Heidl AJ, Angeles PM, Farnesi B-C, Alberga AS, Cohen TR. Assessment of electronic patient education materials for adolescent bariatric surgery candidates: An environment scan. PEC Innov. 2023;2:100143. doi: 10.1016/j.pecinn.2023.100143 37214509 PMC10194287

[43] Meleo-Erwin Z, Basch C, Fera J, Ethan D, Garcia P. Readability of online patient-based information on bariatric surgery. Health Promot Perspect. 2019;9(2):156–60. doi: 10.15171/hpp.2019.22 31249804 PMC6588814

[44] Massie PL, Arshad SA, Auyang ED. Readability of American Society of metabolic surgery’s patient information publications. J Surg Res. 2024;293:727–32. 37862852 10.1016/j.jss.2023.09.018

[45] Mahoney ST, Strassle PD, Farrell TM, Duke MC. Does lower level of education and health literacy affect successful outcomes in bariatric surgery? J Laparoendosc Adv Surg Tech. 2019;29(8):1011–5. 10.1089/lap.2018.0806 31107145

[46] Erdogdu UE, Cayci HM, Tardu A, Demirci H, Kisakol G, Guclu M. Health literacy and weight loss after bariatric surgery. Obes Surg. 2019;29(12):3948–53. doi: 10.1007/s11695-019-04060-7 31290109

[47] Lopes Souto D, Lopes Rosado E. Use of carb counting in the dietary treatment of diabetes mellitus. Nutr Hosp. 2010;25(1):18–25. 20204251

[48] Doak CC, Doak LG, Root JH. Teaching patients with low literacy skills. AJN Am J Nurs. 1996;96(12):16M.

[49] Garnweidner-Holme LM, Dolvik S, Frisvold C, Mosdøl A. Suitability assessment of printed dietary guidelines for pregnant women and parents of infants and toddlers from 7 European countries. J Nutr Educ Behav. 2016;48(2):146–51.e1. doi: 10.1016/j.jneb.2015.10.004 26603301

[50] Rhee RL, Von Feldt JM, Schumacher HR, Merkel PA. Readability and suitability assessment of patient education materials in rheumatic diseases. Arthritis Care Res (Hoboken). 2013;65(10):1702–6. doi: 10.1002/acr.22046 23687011

[51] Tian C, Champlin S, Mackert M, Lazard A, Agrawal D. Readability, suitability, and health content assessment of web-based patient education materials on colorectal cancer screening. Gastrointest Endosc. 2014;80(2):284–90. doi: 10.1016/j.gie.2014.01.034 24674352

[52] Yusof NDM, Hamid MRA, Ridhwan MU. Challenges in nutrition education among patients undergoing bariatric surgery: a narrative review. Environ-Behav Proc J. 2024;9(27).

[53] Messiah SE, Sacher PM, Yudkin J, Ofori A, Qureshi FG, Schneider B, et al. Application and effectiveness of eHealth strategies for metabolic and bariatric surgery patients: a systematic review. Digit Health. 2020;6. doi: 10.1177/2055207619898987 32030193 PMC6977226

[54] Krontoft A. How do patients prefer to receive patient education material about treatment, diagnosis and procedures? —A survey study of patients preferences regarding forms of patient education materials; leaflets, podcasts, and video. Open J Nurs. 2021;11(10):809–27.

[55] Schooley B, Singh A, Hikmet N, Brookshire R, Patel N. Integrated digital patient education at the bedside for patients with chronic conditions: observational study. JMIR Mhealth Uhealth. 2020;8(12):e22947. doi: 10.2196/22947 33350961 PMC7785403

[56] Lynch A. “When the honeymoon is over, the real work begins:” Gastric bypass patients’ weight loss trajectories and dietary change experiences. Soc Sci Med. 2016;151:241–9. doi: 10.1016/j.socscimed.2015.12.024 26820572

[57] Li Z, Pan Y, Zhang Y, Qin J, Lei X. Dietary experiences after bariatric surgery in patients with obesity: a qualitative systematic review. Obes Surg. 2022;32(6):2023–34. 35359201 10.1007/s11695-022-06018-8

[58] Conceição EM, Fernandes M, de Lourdes M, Pinto-Bastos A, Vaz AR, Ramalho S. Perceived social support before and after bariatric surgery: association with depression, problematic eating behaviors, and weight outcomes. Eat Weight Disord. 2020;25(3):679–92. doi: 10.1007/s40519-019-00671-2 30859467

[59] Agneessens F, Waege H, Lievens J. Diversity in social support by role relations: a typology. Soc Netw. 2006;28(4):427–41.

[60] Coulman KD, MacKichan F, Blazeby JM, Donovan JL, Owen-Smith A. Patients’ experiences of life after bariatric surgery and follow-up care: a qualitative study. BMJ Open. 2020;10(2):e035013. doi: 10.1136/bmjopen-2019-035013 32034030 PMC7045271

[61] Wallwork A, Tremblay L, Chi M, Sockalingam S. Exploring partners’ experiences in living with patients who undergo bariatric surgery. Obes Surg. 2017;27(8):1973–81. doi: 10.1007/s11695-017-2594-9 28210964

[62] Farnesi B-C, Kaffash K, Cohen TR, Alberga AS. A qualitative exploration on the needs of health care providers working with adolescents who are undergoing bariatric surgery. Obes Pillars. 2023;6:100067. doi: 10.1016/j.obpill.2023.100067 37990654 PMC10661974

